# Emergence of canine parvovirus-2c in domestic cats and adaptive evolution of the NS1 gene: Molecular epidemiology of feline parvoviruses in Northern Vietnam (2022–2025)

**DOI:** 10.14202/vetworld.2026.3415-3431

**Published:** 2026-08-08

**Authors:** Hai Minh Tran, Giang Huong Thi Tran, Thiet Chi Ngo, Amonpun Rattanasrisomporn, Jatuporn Rattanasrisomporn, Hieu Van Dong

**Affiliations:** 1 Graduate Program in Animal Health and Biomedical Sciences, Faculty of Veterinary Medicine, Kasetsart University, Bangkok 10900, Thailand.; 2 Faculty of Veterinary Medicine, Vietnam National University of Agriculture, Hanoi 131000, Vietnam.; 3 Interdisciplinary Program in Genetic Engineering and Bioinformatics, Graduate School, Kasetsart University, Bangkok 10900, Thailand.; 4 Department of Companion Animal Clinical Sciences, Faculty of Veterinary Medicine, Kasetsart University, Bangkok 10900, Thailand.

**Keywords:** adaptive evolution, canine parvovirus-2c, feline panleukopenia virus, molecular epidemiology, NS1 gene, phylogenetic analysis, polymerase chain reaction, Vietnam

## Abstract

**Background and Aim:**

Feline parvovirus (FPV) remains one of the most important viral pathogens affecting domestic cats worldwide and continues to cause substantial morbidity and mortality despite the availability of effective vaccines. Information regarding the molecular epidemiology, full-length *VP2* and *NS1* gene characteristics, evolutionary dynamics, and circulation of canine parvovirus (CPV) variants in cats remains limited in Vietnam. Therefore, this study investigated the epidemiological, molecular, and evolutionary characteristics of feline parvoviruses circulating in domestic cats in Northern Vietnam between 2022 and 2025.

**Materials and Methods:**

A cross-sectional molecular epidemiological study was conducted using 244 rectal swab samples collected from healthy and clinically affected domestic cats in Hanoi, Bac Ninh, Hung Yen, and Ninh Binh provinces during 2022–2025. Viral DNA was detected by conventional polymerase chain reaction targeting the *VP2* gene. Nine representative positive samples were subjected to sequencing of the full-length *VP2* and *NS1* genes. Phylogenetic relationships, nucleotide identity, amino acid substitutions, recombination events, and natural selection profiles were analyzed using established bioinformatics tools. Associations between epidemiological variables and FPV positivity were evaluated using Fisher’s exact test.

**Results:**

Eleven of 244 samples (4.51%) were positive for parvovirus DNA. Cats younger than 12 months exhibited significantly higher infection rates than older cats (p < 0.05), whereas breed, sex, and health status were not significantly associated with viral detection. The *VP2* and *NS1* genes exhibited high nucleotide conservation, with identities ranging from 97.72%–100% and 98.20%–99.90%, respectively. Phylogenetic analyses demonstrated that seven isolates clustered with Asian FPV lineages, whereas two isolates grouped within the CPV-2c lineage, providing molecular evidence of CPV-2c infection in domestic cats. No recombination events were detected in either gene. Evolutionary analyses identified two positively selected sites within the NS1 protein together with numerous negatively selected sites, indicating predominantly purifying selection with localized adaptive evolution.

**Conclusion:**

FPV remains actively circulating among domestic cats in Northern Vietnam, with highly conserved *VP2* and *NS1* genes. The detection of CPV-2c in cats provides evidence of interspecies viral circulation and highlights the importance of integrated molecular surveillance. The observed adaptive evolution within the NS1 protein improves current understanding of parvovirus evolution and provides valuable baseline information for future epidemiological investigations, vaccine monitoring, and disease control strategies.

## INTRODUCTION

Feline parvovirus (FPV), also known as the causative agent of feline distemper [1, 2] or feline panleukopenia [3], is one of the most important viral pathogens affecting domestic cats worldwide. FPV causes a highly contagious disease characterized by high morbidity and mortality, particularly in young kittens. The virus was first recognized in the early 20th century [4] and has remained relatively stable genetically despite its widespread global distribution [5]. Since its initial description, FPV has been reported in numerous regions, including Iran [6], East Africa [7], Spain [8], Vietnam [9], and China [10]. FPV infects both domestic and free-roaming cats and is associated with severe clinical disease. Mortality rates among susceptible kittens may reach 90% [11, 12]. Raccoons and foxes are also recognized as hosts of FPV, whereas the virus generally does not cause clinical disease in domestic dogs [13]. Clinical manifestations of feline panleukopenia commonly include severe depression, vomiting, diarrhea, leukopenia, enteritis, dehydration, and anorexia [14, 15].

FPV belongs to the species *Carnivore protoparvovirus 1* within the family *Parvoviridae*. It is a non-enveloped virus with a linear, single-stranded DNA genome of approximately 5.1 kb [16]. The viral genome contains two open reading frames encoding two non-structural proteins, NS1 and NS2, and two structural proteins, VP1 and VP2 [17]. VP2 is the major capsid protein and plays pivotal roles in host-cell tropism, receptor binding, hemagglu-tination, and the induction of virus-neutralizing antibodies [18]. Owing to its critical biological functions and antigenic importance, the *VP2* gene has been extensively used as the principal molecular marker in phylogenetic, evolutionary, and epidemiological studies of FPV [19–21]. A previous study demonstrated that amino acid substitutions in the VP2 protein, including Met87Leu, Ile101Thr, Ala300Gly, Asp305Tyr, and Val555Ile, contribute to host range determination and viral adaptation [22]. Likewise, substitution at residue 101 in field FPV isolates has been reported to alter the molecular conformation of the receptor-binding region, thereby affecting viral affinity for the feline transferrin receptor [19]. Additional substitutions, including A91S and T425A, have been associated with altered antigenic properties through reduced recognition by neutralizing antibodies targeting antigenic site A, potentially facilitating evasion of host immune surveillance [20].

In contrast to VP2, the non-structural protein NS1 is essential for viral DNA replication, genome packaging, transcriptional regulation, cytotoxicity, and pathogenicity [23, 24]. Consequently, analysis of the *NS1* gene can provide complementary information regarding viral evolution, adaptation, and selection pressure. However, compared with the *VP2* gene, the molecular evolution of the *NS1* gene has received considerably less attention [20, 25]. Although the *VP2* and *NS1* gene sequences are generally highly conserved among FPV isolates, naturally occurring mutations continue to accumulate and may contribute to viral adaptation, host switching, and genetic diversification [20, 26, 27]. Therefore, simultaneous characterization of the *VP2* and *NS1* genes provides a more comprehensive understanding of FPV evolution, molecular epidemiology, and potential genetic changes associated with viral pathogenicity.

Studies published since 2020 have confirmed the continued circulation of FPV throughout Asia. Molecular detection has been reported in domestic cats with gastrointestinal disease in Japan [28], diarrheic cats in Vietnam [29], and domestic cat populations in China, where widespread viral circulation has been documented [10, 30]. More recently, FPV has also been identified in domestic cats in Indonesia [31]. In addition to point mutations, recombination and natural selection have been recognized as important evolutionary mechanisms contributing to the genetic diversity of FPV and canine parvovirus (CPV) in several countries, including Japan and China [32–34]. Previous studies have demonstrated that recombination involving the *VP2* and *NS1* genes may generate novel viral variants with altered biological characteristics [20]. These evolutionary processes may influence viral virulence, host range, antigenicity, and vaccine effectiveness, highlighting the importance of continuous molecular surveillance and evolutionary investigations.

Despite substantial advances in understanding FPV epidemiology, several important knowledge gaps remain. Most previous investigations have focused primarily on the *VP2* gene because of its importance in viral classification and antigenic characterization, whereas comprehensive analyses of the *NS1* gene, particularly its evolutionary dynamics and selection pressure in naturally circulating field strains, remain limited. Furthermore, although natural CPV-2c infection in domestic cats has been reported in Southeast Asia, including Thailand [35], information regarding its occurrence and molecular characteristics in Vietnam remains scarce. To date, no study in Vietnam has comprehensively combined full-length *VP2* and *NS1* gene characterization with phylogenetic analysis, recombination detection, and natural selection analysis to investigate the molecular evolution of parvoviruses circulating in domestic cats. Considering the close coexistence of cat and dog populations and variable vaccination coverage, Vietnam provides a favorable ecological setting for viral transmission, cross-species circulation, and the genetic evolution of feline and canine parvoviruses. Addressing these knowledge gaps is essential for improving understanding of parvovirus evolution and supporting evidence-based surveillance, vaccination, and disease control strategies.

This study aimed to investigate the epidemiological, molecular, and evolutionary characteristics of parvoviruses circulating in domestic cats in Northern Vietnam between 2022 and 2025. Specifically, the study aimed to characterize the full-length *VP2* and *NS1* gene sequences, evaluate their genetic diversity and phylogenetic relationships, identify amino acid substitutions in the encoded VP2 and NS1 proteins, assess potential recombination events and patterns of natural selection, and investigate epidemiological factors associated with FPV detection. It was hypothesized that parvoviruses circulating in domestic cats in Northern Vietnam exhibit measurable genetic diversity, comprise distinct FPV- and CPV-related lineages, and display evolutionary signatures associated with viral adaptation and potential interspecies circulation.

## MATERIALS AND METHODS

### Ethical approval

The study protocol was reviewed and approved by the Committee on Animal Research and Ethics, Vietnam National University of Agriculture, Hanoi, Vietnam (approval no. CARE-2022/08). All experimental procedures were conducted in accordance with the institutional guidelines for the ethical care and use of animals in research and complied with internationally accepted principles for veterinary biomedical research involving client-owned animals.

This investigation was based exclusively on clinical rectal swab specimens collected from privately owned domestic cats presented to participating veterinary clinics for routine clinical examination or diagnostic evaluation. Sample collection was performed by licensed veterinarians or trained veterinary personnel using standard aseptic procedures designed to minimize discomfort, stress, and the risk of injury. No animal was euthanized, experimentally infected, restrained beyond routine clinical practice, or subjected to additional invasive procedures solely for research purposes.

Before enrolment, cat owners received a detailed explanation of the study objectives, sampling procedures, potential risks, anticipated benefits, and intended use of the collected samples. Written informed consent was obtained from all owners before sample collection. Participation was entirely voluntary, and owners retained the right to withdraw their animals from the study at any stage without affecting the veterinary care provided.

All animal records and owner information were treated as strictly confidential. Personal identifiers were removed before data analysis, and all biological samples and associated data were used exclusively for the purposes described in the approved research protocol. Laboratory procedures involving infectious materials were conducted in accordance with institutional biosafety regulations and established laboratory quality-control practices to ensure personnel safety and maintain sample integrity. This study adhered to the principles of animal welfare, scientific integrity, and responsible conduct of research throughout all stages of sample collection, laboratory analysis, data interpretation, and reporting.

### Study period and location

The study was conducted from May 20, 2022, to December 25, 2025. Sample collection was performed at 12 veterinary clinics located in Hanoi and the provinces of Bac Ninh, Hung Yen, and Ninh Binh in Northern Vietnam. Laboratory analyses were conducted at the Faculty of Veterinary Medicine, Vietnam National University of Agriculture, Hanoi, Vietnam, and the Faculty of Veterinary Medicine, Kasetsart University, Bangkok, Thailand.

### Study design and sample population

**Figure 1:**
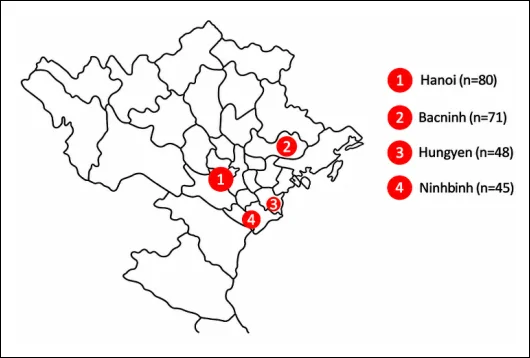
Locations of the city and three provinces selected for sample collection in Northern Vietnam [Source: https:// tomau.vn/].

A cross-sectional molecular epidemiological study was conducted using a convenience sampling approach. A total of 244 rectal swab samples were collected from domestic cats aged 3–36 months that were presented to veterinary clinics in Hanoi (four clinics), Bac Ninh (three clinics), Hung Yen (three clinics), and Ninh Binh (two clinics), Northern Vietnam, between 2022 and 2025 (Figure 1).

Both apparently healthy cats and cats showing clinical signs were included. Cats were classified as sick when they exhibited at least one clinical sign consistent with parvoviral infection during clinical examination, including fever, vomiting, diarrhea, lethargy, or anorexia. Samples with insufficient volume or incomplete clinical information were excluded from the study. Limited follow-up sampling was attempted in a subset of cases; however, repeated sampling could not be performed consistently because of logistical constraints and loss to follow-up.

### Sample collection and processing

Rectal samples were collected using sterile cotton swabs, which were immediately placed in tubes containing 1 mL of phosphate-buffered saline (PBS, pH 7.4). The samples were maintained at 4°C during transport to the Faculty of Veterinary Medicine, Vietnam National University of Agriculture, and processed within 24–48 h of collection. Each sample was vortexed and centrifuged at 1,500 rpm for 5 min. The resulting supernatant was collected and stored at −80°C until molecular analysis.

### DNA extraction and polymerase chain reaction (PCR) detection of the parvovirus genome

Viral DNA was extracted using the Viral Gene-spin™ Viral DNA/RNA Extraction Kit (iNtRON Biotechnology Inc., Seongnam, Republic of Korea) according to the manufacturer’s instructions.

Conventional PCR was performed using a primer pair specific to the parvovirus *VP2* gene. Each PCR was conducted in a final reaction volume of 25 µL containing 12.5 µL of 2× GoTaq™ Green Master Mix (Promega Corporation, Madison, WI, USA), 1 µL each of the forward and reverse primers at a concentration of 10 µM, 8.5 µL of sterile distilled water, and 2 µL of the extracted DNA template. The FPV-F and FPV-R primers used for FPV detection were selected based on previously published studies [20, 25, 29, 36] (Table 1).

The amplification conditions consisted of an initial denaturation at 95°C for 5 min, followed by 35 cycles of denaturation at 95°C for 30 s, annealing at 60°C for 1 min, and extension at 72°C for 1 min, with a final extension at 72°C for 10 min. The PCR products were separated by electrophoresis on a 1.5% agarose gel and visualized using an ultraviolet transilluminator. DNA extracted from the FELOCELL vaccine (Zoetis Inc., Lincoln, NE, USA) served as the positive control, whereas sterile distilled water served as the negative control.

**Table 1:** Primers used for parvovirus detection and nucleotide sequencing.

**Purpose**	**Primer name**	**Sequence (5′–3′)**	**Amplicon size**	**Reference**
Detection	FPV-F	TGC CTC AAT CTG AAG GAG CT	225 bp	[36]
	FPV-R	TTT CAT CTG TTT GCG CTC CC		
Nucleotide sequencing	FPV-S1-F	CCA ACT AAA AGA AGT AAA CC	708 bp	[25]
	FPV-S1-R	TGG TTG GTT TCC ATG GAT AAA AAC C		
	FPV-S2-F	AGA TAG TAA TAA TAC GCC ATT T	719 bp	
	FPV-S2-R	TTT TGA ATC CAA TCT CCT TCT GGA T		
	FPV-S3-F	ACA GGA GAA ACA CCT GAG AGA TTT A	569 bp	[25]
	FPV-S3-R	AGG TGC TAG TTG AGA TTT TTC AT		[29]
	FPV-NS1-1-F	GAC CGT TAC TGA CAT TCG C	881 bp	[20]
	FPV-NS1-1-R	CTT TGT TTC CTG TGC TGT CG		
	FPV-NS1-2-F	GTC CAC ATG ACA AAA GAA AG	1,200 bp	
	FPV-NS1-2-R	CGG CGT CAG AAG GGT T		

### Amplification and nucleotide sequencing of the *VP2* and *NS1* genes

Parvovirus-positive samples were randomly selected for amplification and sequencing of the full-length *VP2* and *NS1* genes. Five primer pairs were used: three primer pairs targeting the *VP2* gene (FPV-S1-F/R, FPV-S2-F/R, and FPV-S3-F/R) and two primer pairs targeting the *NS1* gene (FPV-*NS1*-1-F/R and FPV-*NS1*-2-F/R) (Table 1).

Each PCR was performed in a final volume of 25 µL containing 12.5 µL of GoTaq™ Green Master Mix (Promega Corporation), 1 µL of each primer, 2 µL of the DNA template, and 8.5 µL of sterile distilled water. The amplification conditions consisted of an initial denaturation at 95°C for 5 min, followed by 40 cycles of denaturation at 95°C for 30 s, annealing at 52°C for 30 s, and extension at 72°C for 30 s, with a final extension at 72°C for 10 min.

The PCR products were submitted to 1st BASE, Selangor, Malaysia, for Sanger sequencing. Raw chromatograms were manually inspected, and low-quality nucleotide positions were trimmed based on their Phred quality scores prior to sequence assembly. Full-length *VP2* and *NS1* gene sequences were assembled from overlapping sequence fragments using BioEdit version 7.2.5.

The *VP2* and *NS1* gene sequences obtained from each parvovirus strain were submitted individually to GenBank using the National Center for Biotechnology Information BankIt submission system. The sequences were deposited in GenBank under accession numbers PX761464–PX761481.

### Genetic and phylogenetic analyses

The nucleotide sequences were aligned using ClustalW implemented in BioEdit version 7.2.5 [37]. Pairwise nucleotide identity was determined using GENETYX version 10.0 (GENETYX Corporation, Tokyo, Japan) and the Basic Local Alignment Search Tool program available from the National Center for Biotechnology Information.

Phylogenetic trees were constructed using the full-length *VP2* and *NS1* gene sequences generated in this study and reference sequences retrieved from the GenBank database (Supplementary Tables 1 and 2). Molecular Evolutionary Genetics Analysis version 7.0 was used for phylogenetic reconstruction. The trees were generated using the maximum-likelihood method and the best-fitting Tamura three-parameter nucleotide substitution model. The reliability of the inferred tree topologies was evaluated using bootstrap analysis with 1,000 replicates.

### Recombination and natural selection analyses

Potential recombination events involving the *VP2* and *NS1* gene sequences were investigated using Recombination Detection Program version 5.0 [38]. The analyses were performed using the BootScan, Chimaera, GENECONV, RDP, MaxChi, LARD, SiScan, 3Seq, and PhylPro algorithms with default parameters. Recombination signals were considered statistically significant at p < 0.05. The use of multiple complementary algorithms was intended to improve the robustness of recombination detection and reduce the likelihood of method-specific false-positive findings.

Natural selection profiles were evaluated using the Datamonkey web server and the Fast Unconstrained Bayesian AppRoximation method [39]. The general time-reversible nucleotide substitution model was selected as the best-fitting evolutionary model for the analyzed sequence datasets.

### Integrated molecular and epidemiological approach

The study combined molecular detection, nucleotide sequencing, phylogenetic reconstruction, recombi-nation analysis, natural selection analysis, and supporting epidemiological data to characterize parvoviruses circulating among domestic cats in Northern Vietnam. Limited longitudinal sampling was also attempted in a subset of cases for which follow-up samples could be obtained. However, repeated sampling was not consistently achieved due to logistical constraints and loss to follow-up.

### Statistical analysis

Statistical analyses were performed using Statistical Package for the Social Sciences version 25.0 (IBM Corporation, Armonk, NY, USA). Fisher’s exact test was used for univariate analysis of the associations between FPV positivity and age (<6, 6–12, and >12 months), sex (male and female), breed group (native and exotic or crossbred cats), and health status (healthy and sick).

Multivariable analysis was not performed because of the limited number of parvovirus-positive samples. Statistical significance was defined as p < 0.05.

## RESULTS

### Detection of parvovirus DNA by conventional PCR

Conventional PCR detected parvovirus DNA in 11 of the 244 rectal swab samples collected from domestic cats in Hanoi and the provinces of Bac Ninh, Hung Yen, and Ninh Binh, corresponding to an overall positivity rate of 4.51% (95% confidence interval [CI] = 2.27%–7.92%) (Table 2). Hung Yen had the highest positivity rate at 10.42% (5/48; 95% CI = 3.47%–22.66%), followed by Bac Ninh at 4.23% (3/71; 95% CI = 0.88%–11.86%). Lower positivity rates were detected in Hanoi at 2.50% (2/80; 95% CI = 0.30%–8.74%) and Ninh Binh at 2.22% (1/45; 95% CI = 0.06%–11.77%).

### Epidemiological factors associated with parvovirus detection

Parvovirus positivity was evaluated according to breed, age, sex, and health status (Table 3). Exotic and crossbred cats had a higher positivity rate than native cats, at 4.90% (10/204; 95% CI = 2.38%–8.83%) and 2.50% (1/40; 95% CI = 0.06%–13.16%), respectively; however, the difference was not statistically significant (p = 0.41).

**Table 2:** Detection of parvovirus DNA in field samples using PCR.

**No.**	**City/province**	**No. of samples tested**	**No. of PCR-positive samples**	**Positive rate (%)**	**95% CI**
1	Hanoi	80	2	2.50	0.30–8.74
2	Bac Ninh	71	3	4.23	0.88–11.86
3	Hung Yen	48	5	10.42	3.47–22.66
4	Ninh Binh	45	1	2.22	0.06–11.77
Total		244	11	4.51	2.27–7.92

CI = Confidence interval; PCR = Polymerase chain reaction.

Age was significantly associated with parvovirus detection (p = 0.001). Cats younger than 6 months had the highest positivity rate at 16.67% (4/24; 95% CI = 4.74%–37.38%), followed by cats aged 6–12 months at 12.50% (6/48; 95% CI = 4.73%–25.25%). In contrast, only 0.58% of cats older than 12 months were positive (1/172; 95% CI = 0.01%–3.20%).

No significant association was observed between sex and parvovirus positivity (p = 0.49). Male and female cats had positivity rates of 4.80% (6/125; 95% CI = 1.78%–10.15%) and 4.20% (5/119; 95% CI = 1.38%–9.53%), respectively. Sick cats had a higher positivity rate than apparently healthy cats, at 5.95% (10/168; 95% CI = 2.89%–10.67%) and 1.32% (1/76; 95% CI = 0.03%–7.11%), respectively; however, the difference was not statistically significant (p = 0.21). All 11 parvovirus-positive cats had no reported history of FPV vaccination.

**Table 3:** Parvovirus DNA detection according to breed, age, sex, and health status.

**Variable**	**Category**	**No. of samples tested**	**No. of PCR-positive samples**	**Positive rate (%)**	**95% CI**	**p-value**
Breed	Native cats	40	1	2.50	0.06–13.16	0.41
	Exotic and crossbred cats	204	10	4.90	2.38–8.83	
Age (months)	<6	24	4	16.67ᵃ	4.74–37.38	0.001
	6–12	48	6	12.50ᵃ	4.73–25.25	
	>12	172	1	0.58ᵇ	0.01–3.20	
Sex	Male	125	6	4.80	1.78–10.15	0.49
	Female	119	5	4.20	1.38–9.53	
Health status	Healthy	76	1	1.32	0.03–7.11	0.18
	Sick	168	10	5.95	2.89–10.67	

Different superscript letters within a variable indicate a significant difference between categories (p < 0.05). CI = Confidence interval; FPV = Feline panleukopenia virus; PCR = Polymerase chain reaction.

### Genetic and phylogenetic characteristics of Vietnamese parvovirus strains

**Full-length VP2 gene sequences:** Nine representative parvovirus-positive samples from Northern Vietnam were successfully subjected to full-length *VP2* gene sequencing. Two additional positive samples were excluded from subsequent analyses because of weak amplification or poor-quality sequencing chromatograms. The nine sequenced strains were designated Cat/Vietnam/VNUA-66, Cat/Vietnam/VNUA-73, Cat/Vietnam/VNUA-99, Cat/ Vietnam/VNUA-129, Cat/Vietnam/VNUA-130, Cat/Vietnam/VNUA-180, Cat/Vietnam/VNUA-181, Cat/Vietnam/ VNUA-182, and Cat/Vietnam/VNUA-183. Each full-length *VP2* gene sequence was 1,755 nucleotides long, and no insertions or deletions were detected.

Pairwise nucleotide identity among the nine Vietnamese *VP2* gene sequences ranged from 97.72% to 100% (Table 4). The lowest identity, 97.72%, was observed between VNUA-66 and VNUA-129 or VNUA-130. Complete nucleotide identity was observed between VNUA-73 and VNUA-99, between VNUA-73 or VNUA-99 and VNUA-180, between VNUA-129 and VNUA-130, and among VNUA-181, VNUA-182, and VNUA-183.

**Figure 2:**
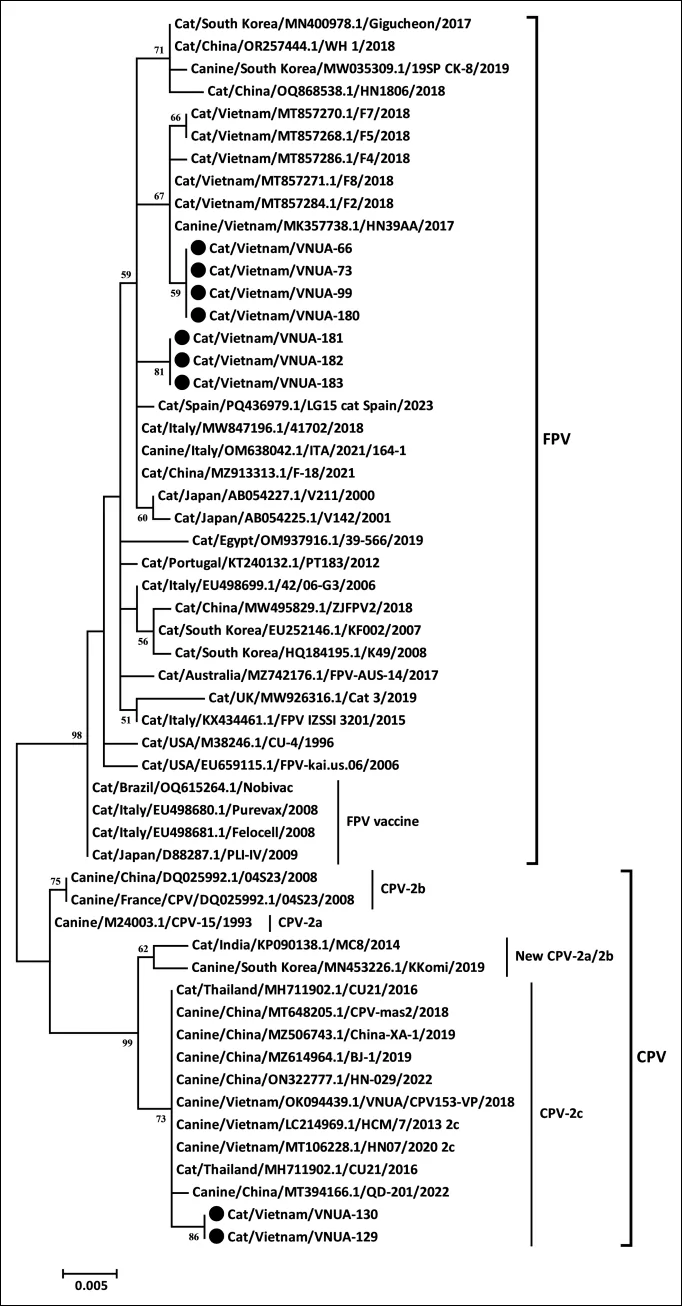
Maximum-likelihood phylogenetic tree based on the full-length *VP2* gene sequences of Vietnamese parvovirus strains and reference sequences retrieved from GenBank. The tree was constructed using Molecular Evolutionary Genetics Analysis version 7.0 with 1,000 bootstrap replicates. Bootstrap values ≥50% are shown at the branch nodes. Vietnamese strains are indicated by solid black circles, and vaccine strains are indicated by the designated vaccine symbols.

**Phylogenetic analysis of the VP2 gene:** The maximum-likelihood phylogenetic tree based on the full-length *VP2* gene sequences separated the Vietnamese strains into two major lineages corresponding to FPV and CPV (Figure 2). Seven strains, VNUA-66, VNUA-73, VNUA-99, VNUA-180, VNUA-181, VNUA-182, and VNUA-183, clustered with previously reported FPV strains from Vietnam, China, and Japan. These isolates formed a well-supported Asian FPV clade with a bootstrap value of 92 and were genetically distinct from the Nobivac, Purevax, CU-4, and PLI-IV vaccine strains.

In contrast, VNUA-129 and VNUA-130 clustered within the CPV-2c lineage together with canine CPV-2c strains from Vietnam, China, and Thailand, with a bootstrap value of 96. Their placement within the CPV-2c lineage demonstrated close genetic relatedness between the feline-derived Vietnamese strains and canine CPV-2c strains circulating in Asia. This finding is consistent with either cross-species transmission or shared circulation of CPV-2c between cats and dogs; however, the direction of transmission could not be determined from the available data.

Comparison with GenBank reference sequences showed that the Vietnamese strains shared 99.59%–99.94% nucleotide identity with their closest reference strains (Table 5). VNUA-73 and VNUA-99 showed the highest identity, at 99.94%, with canine strain HN39AA from Vietnam. VNUA-182 and VNUA-183 showed 99.59% identity with canine strain 164-1 from Italy.

**Table 4:** Pairwise nucleotide identity of the full-length VP2 gene sequences of Vietnamese parvovirus strains.

**Strain**	**VNUA-66**	**VNUA-73**	**VNUA-99**	**VNUA-129**	**VNUA-130**	**VNUA-180**	**VNUA-181**	**VNUA-182**	**VNUA-183**
Cat/Vietnam/PX761464/VNUA-66	100								
Cat/Vietnam/PX761465/VNUA-73	99.82	100							
Cat/Vietnam/PX761466/VNUA-99	99.82	100	100						
Cat/Vietnam/PX761467/VNUA-129	97.72	97.77	97.77	100					
Cat/Vietnam/PX761468/VNUA-130	97.72	97.77	97.77	100	100				
Cat/Vietnam/PX761469/VNUA-180	99.82	100	100	97.77	97.77	100			
Cat/Vietnam/PX761470/VNUA-181	99.77	99.60	99.60	97.72	97.72	99.60	100		
Cat/Vietnam/PX761471/VNUA-182	99.77	99.60	99.60	97.72	97.72	99.60	100	100	
Cat/Vietnam/PX761472/VNUA-183	99.77	99.60	99.60	97.72	97.72	99.60	100	100	100

**Table 5:** Closest GenBank matches of the full-length VP2 gene sequences of Vietnamese parvovirus strains.

**Vietnamese strain**	**GenBank accession number**	**Reference strain**	**Host**	**Country**	**Year**	**Nucleotide identity (%)**
Cat/Vietnam/PX761464/VNUA-66	MK357738.1	HN39AA	Dog	Vietnam	2017	99.77
Cat/Vietnam/PX761465/VNUA-73	MK357738.1	HN39AA	Dog	Vietnam	2017	99.94
Cat/Vietnam/PX761466/VNUA-99	MK357738.1	HN39AA	Dog	Vietnam	2017	99.94
Cat/Vietnam/PX761467/VNUA-129	MH711902.1	CU21	Cat	Thailand	2016	99.77
Cat/Vietnam/PX761468/VNUA-130	MH711902.1	CU21	Cat	Thailand	2016	99.77
Cat/Vietnam/PX761469/VNUA-180	MK357738.1	HN39AA	Dog	Vietnam	2017	99.77
Cat/Vietnam/PX761470/VNUA-181	MW847196.1	41702	Cat	Italy	2018	99.70
Cat/Vietnam/PX761471/VNUA-182	OM638042.1	164-1	Dog	Italy	2021	99.59
Cat/Vietnam/PX761472/VNUA-183	OM638042.1	164-1	Dog	Italy	2021	99.59

**Amino acid substitutions in the VP2 protein:** The predicted amino acid sequences of the VP2 proteins from the nine Vietnamese isolates were compared with those of previously reported FPV field and vaccine strains (Table 6). Thirteen variable amino acid positions were included in the comparison. The VP2 sequences of seven Vietnamese FPV strains were identical to the consensus sequence at all 13 positions. In contrast, VNUA-129 and VNUA-130 contained substitutions at residues 5, 13, 80, 87, 103, 232, 297, 300, 305, 323, 370, and 426, consistent with their classification within the CPV-2c lineage.

**Table 6:** Amino acid substitution profile of the VP2 protein in Vietnamese parvovirus strains.

**Strain**	**5**	**13**	**80**	**87**	**101**	**103**	**232**	**297**	**300**	**305**	**323**	**370**	**426**
**Consensus**	A	P	K	M	T	V	V	S	A	D	D	Q	N
**Cat/Vietnam/PX761464/VNUA-66**	.	.	.	.	.	.	.	.	.	.	.	.	.
**Cat/Vietnam/PX761465/VNUA-73**	.	.	.	.	.	.	.	.	.	.	.	.	.
**Cat/Vietnam/PX761466/VNUA-99**	.	.	.	.	.	.	.	.	.	.	.	.	.
**Cat/Vietnam/PX761467/VNUA-129**	G	S	R	L	.	A	I	A	G	Y	N	R	E
**Cat/Vietnam/PX761468/VNUA-130**	G	S	R	L	.	A	I	A	G	Y	N	R	E
**Cat/Vietnam/PX761469/VNUA-180**	.	.	.	.	.	.	.	.	.	.	.	.	.
**Cat/Vietnam/PX761470/VNUA-181**	.	.	.	.	.	.	.	.	.	.	.	.	.
**Cat/Vietnam/PX761471/VNUA-182**	.	.	.	.	.	.	.	.	.	.	.	.	.
**Cat/Vietnam/PX761472/VNUA-183**	.	.	.	.	.	.	.	.	.	.	.	.	.
Previous Vietnamese FPV	.	.	.	.	.	.	.	.	.	.	.	.	.
Cat/Brazil/OQ615264.1/ Nobivac	.	.	.	.	.	.	I	.	.	.	.	.	.
Cat/Italy/EU498680.1/ Purevax /2008	.	.	.	.	I	.	I	.	.	.	.	.	.
Cat/Italy/EU498681.1/ Felocell /2008	.	.	.	.	.	.	I	.	.	.	.	.	.
Cat/Japan/D88287.1/PLI-IV/2009	.	.	.	.	.	.	I	.	.	.	.	.	.
Cat/USA/M38246.1/CU-4/1996	.	.	.	.	I	.	.	.	.	.	.	.	.

A period indicates identity with the consensus amino acid. The consensus sequence was generated using GENETYX version 10.0 from 100 reference sequences retrieved from GenBank. FPV = Feline panleukopenia virus.

All nine Vietnamese strains encoded threonine at residue 101. Compared with the consensus sequence, the Purevax and CU-4 vaccine strains encoded isoleucine at residue 101. Several vaccine strains also encoded isoleucine rather than valine at residue 232.

**Full-length NS1 gene sequences:** Pairwise nucleotide identity among the nine full-length *NS1* gene sequences ranged from 98.20% to 100%, indicating a high degree of conservation (Table 7). VNUA-129 and VNUA-130 shared complete nucleotide identity. Complete identity was also observed between VNUA-181 and VNUA-183. The lowest identity, 98.20%, was observed between VNUA-182 and VNUA-129 or VNUA-130.

Comparison with GenBank reference sequences showed that the Vietnamese *NS1* gene sequences shared 99.40%–99.80% nucleotide identity with their closest Chinese reference strains (Table 8). The highest identity was observed for VNUA-180, which shared 99.80% identity with feline strain YZ2019-027 from China. VNUA-182 showed the lowest identity (99.40%) with the same reference strain. VNUA-129 and VNUA-130 each shared 99.65% identity with canine CPV-SH1516 from China.

**Table 7:** Pairwise nucleotide identity of the full-length NS1 gene sequences of Vietnamese parvovirus strains.

**Strain**	**VNUA-66**	**VNUA-73**	**VNUA-99**	**VNUA-180**	**VNUA-129**	**VNUA-130**	**VNUA-181**	**VNUA-182**	**VNUA-183**
Cat/Vietnam/PX761473/VNUA-66	100								
Cat/Vietnam/PX761474/VNUA-73	99.50	100							
Cat/Vietnam/PX761475/VNUA-99	99.90	99.50	100						
Cat/Vietnam/PX761476/VNUA-180	99.55	99.85	99.55	100					
Cat/Vietnam/PX761477/VNUA-129	98.50	98.50	98.45	98.55	100				
Cat/Vietnam/PX761478/VNUA-130	98.50	98.50	98.45	98.55	100	100			
Cat/Vietnam/PX761479/VNUA-181	99.90	99.50	99.85	99.55	98.50	98.50	100		
Cat/Vietnam/PX761480/VNUA-182	99.70	99.25	99.75	99.30	98.20	98.20	99.70	100	
Cat/Vietnam/PX761481/VNUA-183	99.90	99.50	99.85	99.55	98.50	98.50	100	99.70	100

**Figure 3:**
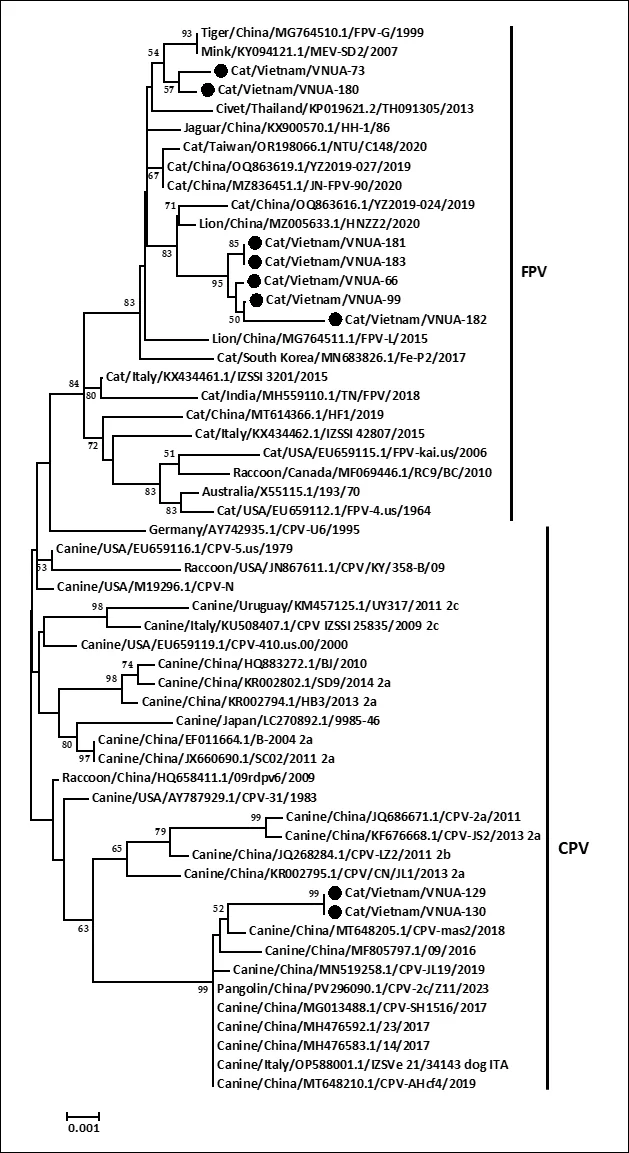
Maximum-likelihood phylogenetic tree based on the full-length *NS1* gene sequences of Vietnamese parvovirus strains and reference sequences retrieved from GenBank. The tree was constructed using Molecular Evolutionary Genetics Analysis version 7.0 with 1,000 bootstrap replicates. Bootstrap values ≥50% are shown at the branch nodes. Vietnamese strains are indicated by solid black circles, and vaccine strains are indicated by the designated vaccine symbols.

**Phylogenetic analysis of the NS1 gene:** Full-length *NS1* sequences separated the Vietnamese strains into two major lineages corresponding to FPV and CPV (Figure 3). Seven strains, VNUA-66, VNUA-73, VNUA-99, VNUA-180, VNUA-181, VNUA-182, and VNUA-183, clustered with previously reported Asian FPV strains. These isolates were further separated into two subclades with bootstrap support values ranging from 83 to 95, indicating genetic diversity among the Vietnamese FPV strains.

VNUA-129 and VNUA-130 clustered within the CPV lineage and were closely related to canine CPV-2c strains from Asian countries, with a bootstrap value of 99. Their consistent placement within the CPV-2c lineage in both the *VP2*- and *NS1*-based trees supports their classification as feline-derived CPV-2c strains.

**Table 8:** Closest GenBank matches of the full-length NS1 gene sequences of Vietnamese parvovirus strains.

**Vietnamese strain**	**GenBank accession number**	**Reference strain**	**Host**	**Country**	**Year**	**Nucleotide identity (%)**
Cat/Vietnam/PX761473/VNUA-66	OQ863619.1	YZ2019-027	Cat	China	2019	99.65
Cat/Vietnam/PX761474/VNUA-73	OQ863619.1	YZ2019-027	Cat	China	2019	99.75
Cat/Vietnam/PX761475/VNUA-99	OQ863619.1	YZ2019-027	Cat	China	2019	99.65
Cat/Vietnam/PX761477/VNUA-129	MG013488.1	CPV-SH1516	Dog	China	2017	99.65
Cat/Vietnam/PX761478/VNUA-130	MG013488.1	CPV-SH1516	Dog	China	2017	99.65
Cat/Vietnam/PX761476/VNUA-180	OQ863619.1	YZ2019-027	Cat	China	2019	99.80
Cat/Vietnam/PX761479/VNUA-181	OQ863616.1	YZ2019-024	Cat	China	2019	99.65
Cat/Vietnam/PX761480/VNUA-182	OQ863619.1	YZ2019-027	Cat	China	2019	99.40
Cat/Vietnam/PX761481/VNUA-183	OQ863619.1	YZ2019-027	Cat	China	2019	99.65

**Amino acid substitutions in the NS1 protein:** Analysis of the predicted NS1 amino acid sequences identified substitutions at residues 52, 60, 248, 346, and 664 among the Vietnamese strains (Table 9). These substitutions comprised Q52H, I60V/L, I248T, L346V/R, and R664Q. The Q52H substitution was detected in VNUA-182, whereas L346V and L346R were detected in VNUA-73 and VNUA-99, respectively. Relative to the Purevax, PLI-IV, and CU-4 vaccine strains, sequence variation was observed at residues 52, 60, 247, 248, 346, and 664.

**Table 9:** Amino acid substitution profile of the NS1 protein in Vietnamese parvovirus strains.

**Strain**	**52**	**60**	**247**	**248**	**346**	**664**
**Consensus**	Q	I	Q	I	L	R
**Cat/Vietnam/PX761473/VNUA-66**	.	V	.	T	.	Q
**Cat/Vietnam/PX761474/VNUA-73**	.	L	.	T	V	Q
**Cat/Vietnam/PX761475/VNUA-99**	.	.	.	T	R	.
**Cat/Vietnam/PX761477/VNUA-129**	.	.	.	.	.	.
**Cat/Vietnam/PX761478/VNUA-130**	.	V	.	.	.	.
**Cat/Vietnam/PX761476/VNUA-180**	.	V	.	T	.	.
**Cat/Vietnam/PX761479/VNUA-181**	.	V	.	T	.	.
**Cat/Vietnam/PX761480/VNUA-182**	H	L	.	T	.	Q
**Cat/Vietnam/PX761481/VNUA-183**	.	V	.	T	.	.
Cat/Italy/EU498680.1/ Purevax /2008	.	.	H	T	.	.
Cat/Japan/D88287.1/PLI-IV/2009	.	.	H	T	.	.
Cat/USA/M38246.1/CU-4/1996	.	.	H	T	.	.

A period indicates identity with the consensus amino acid. The consensus sequence was generated using GENETYX version 10.0 from 100 reference sequences retrieved from GenBank.

### Evolutionary characteristics of the Vietnamese parvovirus strains

**Recombination analysis:** Recombination analysis was performed using the full-length *VP2* and *NS1* gene sequences of the Vietnamese strains and reference sequences retrieved from GenBank. No statistically supported recombination events were detected in either the *VP2* or *NS1* gene dataset using Recombination Detection Program version 5.

**Natural selection analysis:** Natural selection analysis identified two positively selected codons in the NS1 protein, located at amino acid positions 60 and 664 (Table 10). In addition, 30 negatively selected sites were identified in the NS1 protein and 19 negatively selected sites were identified in the VP2 protein (Supplementary Tables 3 and 4). These findings indicated that both proteins were predominantly subject to purifying selection, with localized evidence of adaptive selection in NS1.

**Table 10:** Natural selection profile of the NS1 protein sequences of Vietnamese parvovirus strains

**No.**	**Site**	**Partition**	**α**	**β**	**β − α**	**Posterior probability (α > β)**	**Posterior probability (α < β)**	**Bayes factor (α < β)**
1	60	1	2.545	25.476	22.931	0.024	0.950	28.760
2	664	1	2.072	28.911	26.839	0.013	0.968	45.904

α = Synonymous substitution rate; β = Nonsynonymous substitution rate. Sites with β > α and a high posterior probability were interpreted as being under positive selection.

## DISCUSSION

### Overall findings

This study detected parvovirus DNA in 4.51% of rectal swab samples collected from domestic cats in Northern Vietnam. Rectal swabs were selected as the primary specimens because FPV is shed at high concentrations in feces and this sample type is practical for field-based molecular surveillance. Nine full-length *VP2* and *NS1* gene sequences were successfully obtained and characterized. The Vietnamese parvovirus strains exhibited high nucleotide identity, ranging from 97.72% to 100% for the *VP2* gene and from 98.20% to 99.90% for the *NS1* gene. Phylogenetic analyses showed that seven strains belonged to the FPV lineage, whereas two feline-derived strains clustered within the CPV-2c lineage. No recombination events were detected in either the *VP2* or *NS1* gene dataset. Two positively selected sites were identified in the NS1 protein, together with numerous negatively selected sites in the NS1 and VP2 proteins, indicating predominantly purifying selection with localized adaptive evolution.

### Parvovirus detection and epidemiological implications

The overall parvovirus positivity rate in the present study was 4.51%, as determined by conventional PCR analysis of rectal swabs collected from domestic cats in Northern Vietnam. This rate was lower than the prevalence rates of 19.2% [40] and 65.8% [41] reported in China, 43% reported in Egypt [42], and 73.5% reported in Southern Italy [43]. These differences may reflect variations in the sampled populations, clinical status of enrolled cats, geographic distribution, sample size, specimen type, vaccination coverage, and diagnostic procedures. In particular, the inclusion of both apparently healthy and clinically affected cats, rather than restricting sampling to cats with clinical signs consistent with feline panleukopenia, may have contributed to the relatively low detection rate.

Despite the lower positivity rate, the findings confirm that parvoviruses continue to circulate among domestic cats in Northern Vietnam. Several imported FPV vaccines are available in Vietnam, including FELOCELL 3 (Zoetis) and Purevax feline vaccines (Boehringer Ingelheim Animal Health, Ingelheim am Rhein, Germany); however, vaccination coverage in the general feline population is presumed to remain suboptimal based on personal communication. Previous studies have demonstrated that FPV infection is significantly more frequent in unvaccinated cats than in vaccinated cats [44]. Consistent with this observation, all 11 parvovirus-positive cats in the present study had no reported history of FPV vaccination. Although the absence of vaccinated positive cats does not establish a statistically significant association due to the limited number of positive samples and the lack of comprehensive vaccination data for the sampled population, this finding underscores the importance of improving routine feline vaccination coverage.

### Genetic conservation of the *VP2* and *NS1* genes

The nucleotide identity among the Vietnamese parvovirus strains ranged from 97.72% to 100% for the *VP2* gene and from 98.20% to 99.90% for the *NS1* gene. Similarly high nucleotide identities have been reported in studies from China, including 97.60%–100% for the *VP2* gene and 98.20%–100% for the *NS1* gene [45], as well as 98.30%–99.70% for the analyzed parvovirus sequences [46]. Although most molecular investigations of feline parvoviruses have focused on the *VP2* gene, the nucleotide identities reported in studies from Vietnam and Egypt were also comparable with those obtained in the present study, ranging from 98.30% to 100% [29, 47].

Comparisons among FPV, CPV-2, mink parvovirus, and raccoon parvovirus have likewise demonstrated nucleotide identities exceeding 97% in the *VP2* or *NS1* gene sequences [48, 49]. The consistency of these findings across different hosts and geographic regions indicates that both genes are highly conserved [50, 51]. Nevertheless, the observed sequence differences were sufficient to distinguish FPV and CPV lineages and to identify locally circulating variants, demonstrating the value of combining analyses of the full-length *VP2* and *NS1* genes.

### Phylogenetic relationships of Vietnamese FPV strains

The phylogenetic tree based on full-length *VP2* gene sequences showed that seven Vietnamese strains, excluding Cat/Vietnam/VNUA-129 and Cat/Vietnam/VNUA-130, formed a distinct FPV clade. Their close phylogenetic relationship suggests the circulation of a genetically related lineage in Northern Vietnam. These strains were also closely related to Cat/Vietnam/MT857271.1/F8/2018, indicating a possible shared evolutionary origin with previously reported Vietnamese strains. However, F8/2018 cannot be identified as the direct or most recent common ancestor solely on the basis of the present phylogenetic tree. Confirmation would require broader temporal sampling, additional reference sequences, and whole-genome analysis.

Geographically associated FPV lineages have also been reported in China, where locally circulating strains formed distinct phylogenetic clusters [52]. The comparable pattern observed in Northern Vietnam suggests that local transmission networks, host movement, regional animal-trading practices, and vaccination patterns may contribute to the persistence and geographic structuring of FPV populations.

### Amino acid variation in the VP2 protein

Several VP2 amino acid residues, including positions 93, 103, 300, and 323, have been identified as important determinants of host range [53]. Substitutions at positions 87, 101, 300, 305, and 555 have also been proposed to contribute to viral adaptation [22]. In the present study, all feline-derived parvovirus strains possessed threonine at VP2 residue 101, consistent with previous findings from Vietnam [54].

Residue 101 has been associated with transferrin receptor-binding and the antigenic structure of the viral capsid [19]. Its conservation among the Vietnamese strains may therefore reflect structural or functional constraints required for efficient viral attachment or host adaptation. However, because all analyzed strains carried threonine at this position, the present data cannot directly establish the effect of this substitution on receptor affinity, antigenicity, virulence, or host range. Functional studies involving receptor-binding assays, reverse genetics, or virus-neutralization experiments are required to determine the biological consequences of variation at this residue.

### Detection of feline-derived CPV-2c strains

Phylogenetic analysis of the full-length *NS1* gene sequences showed that seven of the nine Vietnamese strains belonged to the FPV lineage and were divided into several subclades. Importantly, analyses of both the *VP2* and *NS1* genes consistently classified Cat/Vietnam/VNUA-129 and Cat/Vietnam/VNUA-130 within the CPV-2c lineage, supporting their identification as feline-derived CPV-2c strains.

Previous research demonstrated that CPV-2c is the predominant CPV variant circulating among dogs in Vietnam, accounting for approximately 97.3% of CPV-positive canine samples in Northern Vietnam [55]. Dogs and cats are also frequently housed together or share household and community environments in Vietnam, potentially increasing their exposure to contaminated feces, fomites, premises, and other environmental sources of parvoviruses [56]. Therefore, the detection of CPV-2c in cats may indicate shared environmental circulation or possible transmission between canine and feline populations [54, 57].

Nevertheless, the current findings do not establish the direction or precise route of transmission. Viral DNA detected in rectal swabs could represent active feline infection, transient intestinal passage, or environmental contamination. Demonstration of productive infection would require additional evidence, such as virus isolation, quantitative assessment of viral load, detection of viral replication in tissues, serological responses, or longitudinal sampling. Despite this limitation, the consistent placement of VNUA-129 and VNUA-130 within the CPV-2c lineage in both gene-based phylogenies provides molecular evidence that cats in Northern Vietnam are exposed to CPV-2c.

These findings emphasize the importance of routine vaccination and integrated molecular surveillance of parvoviruses in both cats and dogs. Continuous monitoring would facilitate the early detection of emerging variants, improve understanding of cross-species circulation, and support evaluation of whether currently available vaccines remain antigenically appropriate for locally circulating strains.

### Recombination patterns and viral evolution

Recombination is an important mechanism contributing to viral diversity and evolution [58]. Previous studies have demonstrated that recombination in feline parvoviruses may involve the *VP2* [59, 60] or *NS1* [61] gene, or both [50]. In contrast, no evidence of recombination was detected in either the *VP2* or *NS1* gene sequences analyzed in the present study.

The absence of detectable recombination suggests that the evolution of the investigated Vietnamese strains was more likely driven by the accumulation of point mutations than by the exchange of large genomic regions. Although recombination is considered relatively infrequent among parvoviruses, point mutations may produce structural or functional changes in key viral proteins, including the VP2 capsid protein and the replication-associated NS1 protein [62]. Such changes may influence host adaptation, immune evasion, replication efficiency, and persistence under diverse environmental conditions, thereby contributing to viral evolution [63, 64]. However, the limited number of analyzed sequences reduced the probability of detecting rare recombination events; therefore, recombination cannot be excluded from the broader Vietnamese parvovirus population.

### Natural selection acting on the NS1 and VP2 proteins

Two positively selected sites were identified in the NS1 protein at amino acid positions 60 and 664, whereas 30 negatively selected sites were identified in NS1 and 19 negatively selected sites were identified in VP2. To our knowledge, this is among the first studies to report positively selected sites in the NS1 protein of field FPV strains circulating in Vietnam.

NS1 contains several functionally important regions, including the origin-of-replication-binding domain at amino acid positions 16–275, the helicase domain at positions 299–486, and the transactivation domain at positions 600–667 [65]. Accordingly, residue 60 is located within the origin-of-replication-binding region, whereas residue 664 is located near the end of the transactivation domain. A nonsynonymous mutation at NS1 residue 60 has previously been reported and may be associated with viral genome replication or virion packaging [20]. The detection of positive selection at residues 60 and 664 may therefore indicate localized adaptive pressure affecting replication-related or transcriptional functions. However, these functional implications remain hypothetical because no experimental assays were conducted to evaluate the biological effects of the identified substitutions.

FPV has been extensively investigated in China and Japan; however, most previous studies have focused on the *VP2* gene, and relatively few have examined the molecular evolution of the *NS1* gene [10, 20, 66, 67]. Available evidence indicates that NS1 evolution is generally dominated by purifying selection, with limited and inconsistent evidence of recombination [20, 67]. The identification of two positively selected NS1 sites without detectable recombination in the Vietnamese strains may reflect localized adaptive evolution superimposed on strong overall functional constraints. Nonetheless, broader sequence datasets from different host species, geographic regions, and collection periods are required to determine whether these sites represent persistent regional selection signals or findings specific to the limited dataset analyzed here.

The predominance of negatively selected sites in both NS1 and VP2 indicates strong purifying selection. Such selection likely preserves functions essential for viral replication and maintains the structural integrity of the capsid. Most Vietnamese strains remained within established FPV lineages, suggesting limited divergence from classical FPV strains and no immediate evidence of extensive antigenic change that would compromise current vaccination programs. However, vaccine effectiveness was not directly assessed in this study, and the detection of feline-derived CPV-2c strains highlights the need for continued monitoring of antigenic and genetic variation.

### Study strengths, limitations, and future directions

A major strength of this study was the integrated analysis of full-length *VP2* and *NS1* gene sequences together with epidemiological data, phylogenetic reconstruction, amino acid comparisons, recombination screening, and natural selection analysis. This approach provided a broader view of parvovirus evolution than studies based exclusively on partial *VP2* gene sequences. The inclusion of samples from one city and three provinces also generated baseline information on circulating parvoviruses across multiple locations in Northern Vietnam.

Several limitations should be considered. Only nine positive samples were successfully sequenced, and CPV-2c was identified in two cats; therefore, conclusions regarding the prevalence and co-circulation of FPV and CPV-2c should be interpreted cautiously. Convenience sampling and recruitment through veterinary clinics may also have introduced selection bias and limited the representativeness of the sampled population. Furthermore, detailed vaccination records, clinical outcomes, viral loads, serological findings, and household contact histories were unavailable for all cats. The study analyzed two full-length genes rather than complete viral genomes, potentially limiting the detection of recombination or adaptive changes occurring elsewhere in the genome. Finally, no virus isolation or functional assays were performed to confirm productive CPV-2c infection in cats or assess the biological effects of the positively selected NS1 sites.

Future studies should include larger, geographically representative populations of owned, free-roaming, and shelter cats, as well as sympatric dog populations. Whole-genome sequencing, quantitative PCR, virus isolation, serological testing, and longitudinal follow-up would help clarify transmission pathways, host adaptation, and the clinical significance of feline-derived CPV-2c. Functional characterization of NS1 residues 60 and 664 is also warranted to determine whether these substitutions affect viral replication, transcriptional activity, pathogenicity, or host range.

## CONCLUSION

This study provides the first comprehensive molecular characterization of feline parvoviruses circulating among domestic cats in Northern Vietnam, based on analyses of the full-length *VP2* and *NS1* genes. Parvovirus DNA was detected in 4.51% of rectal swab samples, confirming that FPV continues to circulate among domestic cats in the region. Genetic analyses demonstrated that the Vietnamese strains were highly conserved, with nucleotide identities ranging from 97.72% to 100% for the *VP2* gene and from 98.20% to 99.90% for the *NS1* gene. Phylogenetic reconstruction showed that seven isolates belonged to the classical FPV lineage, whereas two feline-derived isolates clustered within the CPV-2c lineage, suggesting exposure of cats to canine-associated parvovirus strains circulating in Vietnam. No recombination events were detected in either the *VP2* or *NS1* genes, while natural selection analysis identified two positively selected sites in the NS1 protein and extensive purifying selection across both genes, indicating that point mutations, rather than recombination, are likely the predominant mechanism driving the evolution of these Vietnamese parvovirus strains.

The detection of both FPV and feline-derived CPV-2c strains highlights the importance of continuous molecular surveillance of parvoviruses in both cat and dog populations. The finding that all PCR-positive cats had no reported vaccination history further underscores the need to increase vaccination coverage to reduce viral circulation and disease burden. Regular monitoring of circulating strains will also provide valuable information for evaluating the continued suitability of commercially available vaccines and supporting evidence-based disease control strategies.

Collectively, the findings demonstrate that FPV remains the predominant parvovirus circulating among domestic cats in Northern Vietnam and provide molecular evidence of feline-derived CPV-2c strains. The high genetic conservation of the *VP2* and *NS1* genes, the absence of detectable recombination, and the predominance of purifying selection indicate that the current Vietnamese parvovirus population remains evolutionarily stable despite localized adaptive changes in NS1. These baseline molecular data contribute to a better understanding of FPV evolution in Southeast Asia and provide an important foundation for future surveillance, vaccine evaluation, and integrated control strategies targeting parvovirus infections in companion animals.

## DATA AVAILABILITY

The data generated during the study are included in the manuscript.

## GENERATIVE AI DECLARATION

The authors declare that no generative artificial intelligence or AI-assisted technologies were used in the writing, analysis, or preparation of this manuscript.

## AUTHORS’ CONTRIBUTIONS

HMT, HVD, and JR: Conceptualization, study design, and methodology. HMT, GHTT, TCN, and HVD: Sample collection, laboratory investigation, and data collection. HMT, HVD, and AR: Data analysis, bioinformatics, and data interpretation. HMT, HVD, and JR: Writing – original draft. GHTT, TCN, and AR: Validation and manuscript review and editing. All authors read and approved the final version of the manuscript.
